# RORγt-APCs: The New Masters of Oral Tolerance

**DOI:** 10.1002/dni2.70001

**Published:** 2025-07-22

**Authors:** Thierry Gauthier, WanJun Chen

**Affiliations:** Mucosal Immunology Section, National Institutes of Dental and Craniofacial Research (NIDCR), National Institutes of Health, Bethesda, Maryland, USA

## Abstract

Oral tolerance is defined by the hypo-responsiveness of our body to fed antigens, and its failure can lead to immune-mediated diseases, such as allergy, chronic inflammation and autoimmune diseases. Decades of research have demonstrated that antigen-presenting cells (APCs) promote oral tolerance by inducing regulatory T cells (Tregs) and/or suppressing T cell activation. Nevertheless, the exact subsets of APCs involved in the presentation of antigens to T cells remains under debate. Recent exciting studies have described several subsets of RORγt (Retinoic acid-related orphan receptor gamma t)-APCs that have key roles in regulating immune responses and oral tolerance. In this review, we briefly summarize the current knowledge about oral tolerance development and then focus on discussing how these newly identified RORγt-APCs act as crucial regulators of this process.

## 1 | Introduction

The human digestive system is composed of a group of organs that have for main function to process food and liquids to generate energy. This process must ensure that the digested food antigens are “tolerized” by the mucosal immune system in the gut. Nevertheless, the past decades have shown that the digestive system is constantly in contact, as a mucosal tissue barrier, with billions of microorganisms (bacteria, fungi and/or viruses) [1]. This gut microbiota is mainly composed of symbionts that synergize with the intestinal cells to maintain a balance in our body and promote health. In the meantime, dysbiosis of this microbiota and/or external infectious agents can lead to disease development [2]. Thus, the mucosal system in the gut must, in addition to process food, also ensure the homeostasis of this microbiota and prevent the proliferation/infection of pathogenic microbes. It is therefore crucial for the gut to maintain a proper balance between immune tolerance and immunity [1, 3–5].

An important mechanism that allows to enforce this balance is the oral tolerance to food. The concept of oral tolerance has been described a few decades ago and is defined by the hypo-responsiveness of our body to fed antigens, namely our body does not mount a harmful immune response to antigens exposure via the oral route [6, 7]. It is a natural and crucial mechanism given that we ingest more than 100 g of dietary (foreign) proteins every day. Induction of oral tolerance will result in inhibition of subsequent immune responses to the same antigen, or other antigens through a so-called by-stander suppression [8]. This is vital because a break-down in oral tolerance mechanisms can result in immunological pathologies, such as IgE (Immunoglobulin E)-mediated food allergies, delayed-type hypersensitivity, celiac disease and other autoimmune pathologies [7, 9–16].

Oral tolerance is mediated by the suppression of food antigen-specific T cells. The antigen is taken up inside the intestinal lumen and transported to the lamina propria and the gut-associated lymphoid organs (GALT). Then, the antigens are captured by antigen-presenting cells (APCs) that can process and present them to T cells via MHC (Major Histocompatibility Complex) molecules. This will generally result in the differentiation of these T cells toward a Treg phenotype which will inhibit the mounting of an immune response against these antigens [17, 18]. However, several questions in this field remain unanswered. One that has seen the focus of many studies is which type(s) of APCs is/are responsible for the induction of oral tolerance. Recent advances in developmental studies, generation of mouse models, as well as the advances of cutting-edge sequencing, have shed new insights into this question. Despite the initial view that certain subsets of dendritic cells (DCs) are responsible for the induction of oral tolerance, the recent discovery of several subsets of RORγt-APCs suggests that the picture might be more complex than we first thought [19].

In this review, we will highlight and discuss the recent advances in the field related to the discovery of RORγt-APCs and their regulation in oral tolerance, and then provide our perspective with a focus on the outstanding remaining questions in the field.

## 2 | Mechanisms of Oral Tolerance Establishment

**FIGURE 1 |:**
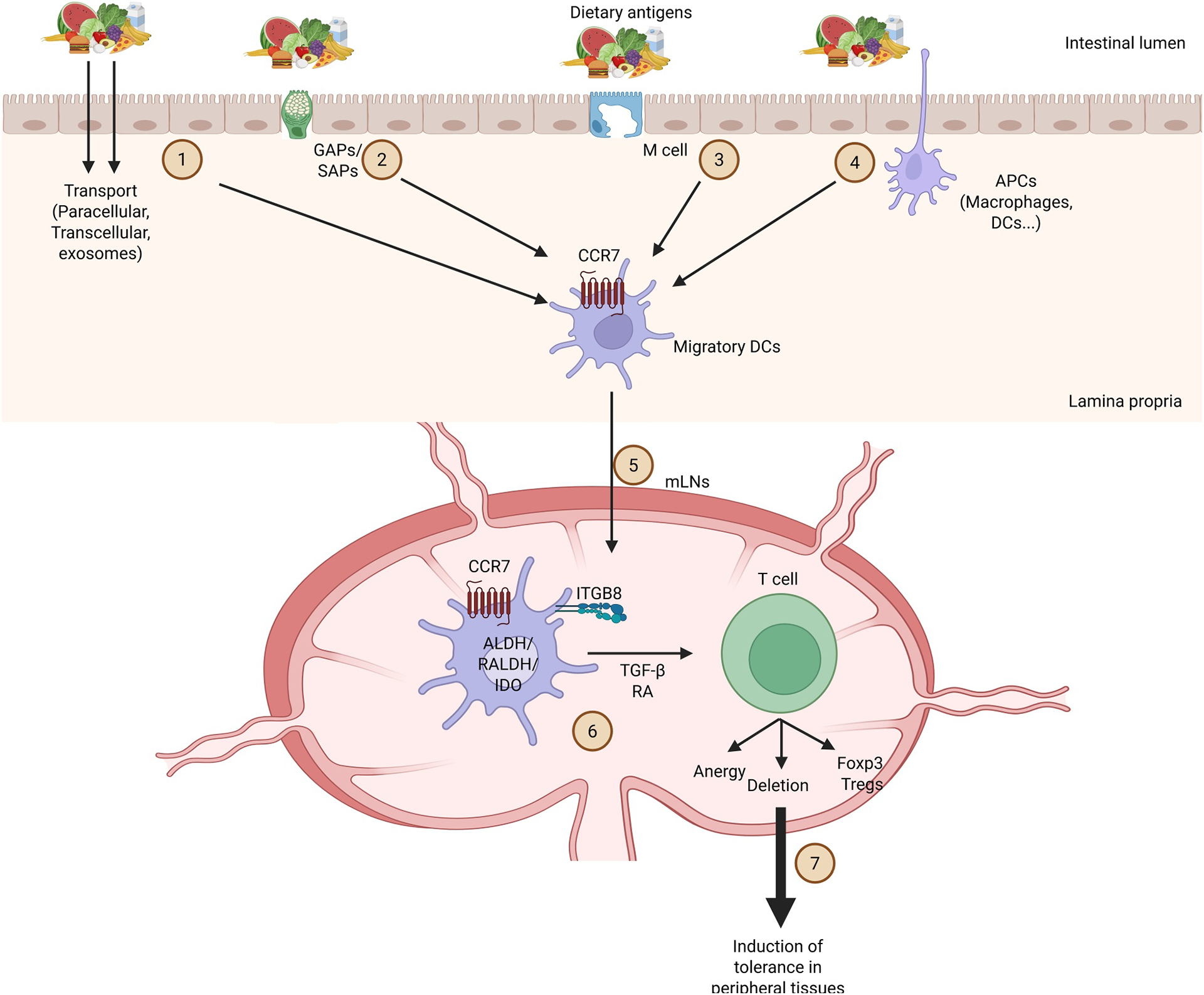
Mechanisms of oral tolerance induction. The induction of oral tolerance starts with the uptake of dietary antigens [1–4] and its transfer to migratory dendritic cells (DCs). The transfer of antigens to DCs can occur via different possible mechanisms such as transport across the epithelium [1], transfer via goblet cells [2], M cells [3] or via the ability of APCs to sense the intestinal lumen [4]. DCs therefore migrate to the mesenteric lymph nodes [5] where they can potently modulate T cells functions [6]. This is notably due to their enhanced capacity in producing/activating RA and TGF-β. The subsequent induction of anergy, deletion of autoreactive T cells and induction of Tregs allows the induction of peripheral tolerance to the fed antigens. ALDH, Aldehyde Dehydrogenase; APCs, antigen-presenting cells; CCR7, C-C Chemokine Receptor type 7; DCs, Dendritic cells; Foxp3, Forkhead box P3; GAPs/SAPs, Goblet/Secretory cell-associated Passages; IDO, Indoleamine 2,3-dioxygenase; ITGB8, Integrin beta-8; mLNs, Mesenteric Lymph Nodes; RA, Retinoic Acid; RALDH, Retinaldehyde Aldehyde Dehydrogenase; TGF-β, Transforming Growth Factor Beta; Tregs, Regulatory T cells.

The first step in oral tolerance induction is the uptake of antigens in the intestine. It is well known that the low pH of the intestine, in combination with several proteolytic enzymes, efficiently degrades the food that we ingest. Nevertheless, some food components are not entirely degraded and can serve as food antigens after crossing the epithelium. Although this is not the focus of this review, it is important to note that despite several interesting hypothesis, it is rather unclear how food antigens are taken up by APCs (Figure 1). A possible explanation lies in the transportation of antigens (such as haptens, peptides or small proteins) across the epithelium, possibly through tight junctions or via exosomes (also called tolerosomes) that are derived from epithelial cells [19, 20]. Another possibility is that goblet cell-associated passages (GAPs) or soluble cell-associated passages (SAPs) could take up soluble antigens from the lumen of the intestine, via endocytosis, and release them, via *trans*-endocytosis, to their basal membrane into the lamina propria. There, APCs could take up these antigens [21, 22]. It has also been suggested that Microfold cells could play a role in this process as well [23]. In addition, it has been postulated that APCs could directly sample the intestinal lumen. Notably, CX3CR1^+^ (CX3C motif chemokine receptor 1) macrophages can send protrusion into the lumen and take up soluble food antigens. These macrophages are then able to transfer the acquired antigens to dendritic cells (DCs), which will then mediate oral tolerance induction [24]. However, which populations of DCs and macrophages are involved in this process is still unclear. Moreover, and as we will discuss later, whether RORγt-APCs are able to sample the intestinal lumen directly remains unknown.

The next step to induce tolerance is the presentation of antigens by APCs to T cells (Figure 1). Several pieces of evidence in the literature show that the DCs are the main APCs subset that induces oral tolerance [25]. This is in part because DCs are naturally professional at presenting antigens due to the natural expression of proteins involved in antigen presentation machinery [26], but also because (and contrary to other subsets of APCs such as macrophages) they are capable to migrate between the lamina propria and the mesenteric lymph nodes (mLNs). Notably, altering the capacity of DCs to migrate to the mLNs (or removing the mLNs) abrogates tolerance [27]. Moreover, it seems that the DCs in intestines have unique phenotypic properties that allows them to direct the differentiation of T cells toward the generation of Tregs. For instance, intestinal DCs produce high levels of TGF-β (Transforming Growth Factor-β) and express its activating factor integrin αvβ8 (ITGB8), which cleaves inactive TGF-β into active TGF-β [12, 28–31]. Moreover, DCs also produce high levels of retinoic acid because of high expression of aldehyde dehydrogenase and retinaldehyde dehydrogenase [29, 32]. The combination of high levels of TGF-β and retinoic acid therefore promotes the generation of Tregs in the gut [33], a core force of inducing and maintaining oral tolerance. It is also to be noted that DCs can express several other factors promoting Treg differentiation such as indoleamine 2,3-dioxygenase (IDO) [34]. The generated antigen-specific Tregs are therefore able to suppress the activation of other T cell subsets (such as T helper 1 (Th1), Th2 and Th17) in the gut and also in distant tissues such as the skin or the lungs [10, 35]. Besides Treg generation, it is also important to note that other mechanisms of oral tolerance induction have been described such as T cell anergy and/or deletion [9, 36].

A possible important factor to regulate oral tolerance induction could be the microbiota. Indeed, it is now well described that the microbiota can dramatically modulate the different functions of the immune system and that changes in microbiota are associated with the development of several autoimmune diseases [37–39]. In addition, the microbiota is a source of antigens and a critical factor to maintain intestinal homeostasis. The microbiota is notably known to regulate both the induction of Tregs (in several tissues) and to regulate their functions [40–42]. It becomes clearer that the microbiota can influence the development of oral tolerance, although the precise mechanisms remain to be fully understood. It was demonstrated early on that depletion of microbiota using the antibiotic erythromycin altered tolerogenic DCs in the mLNs, which impairs the induction of oral tolerance [43]. Similar findings were reported more recently with a combination of kanamycin, metronidazole, colistin and vancomycin [44]. However, another report suggests that the transient depletion of microbiota had an opposite effect, leading to promote the induction of oral tolerance [45]. Interestingly, therapies aimed at rebalancing the microbiota or some of its products such as serotonin can restore the induction of oral tolerance and protect against food allergy [46, 47]. As we will discuss later, the microbiota also seems to have an effect on RORγt-APCs during the establishment of oral tolerance [48, 49].

Nevertheless, it should be noted that a limitation of most oral tolerance studies is that they have been mostly performed in mice, but few other animal models (or human data) have been used. The first reports on oral tolerance were performed on guinea pigs by Besredka, as well as Wells and Osborne at the beginning of the 20th century. They notably showed the resistance of guinea pigs to anaphylaxis induced by milk and corn after induction of oral tolerance [50]. It has also been shown that oral tolerance can be induced in several other species such as dogs, rabbits or rats, although the mechanisms have not been studied in detail [51–53]. Thus, an important task in the field is to translate the studies in animals to human diseases in the future.

An important question that has been the subject of discussion in the past two decades is which type(s) of APCs are responsible for the induction of oral tolerance. Although macrophages seem to play a role in the overall process, they likely are not responsible for the presentation of antigens to T cells [24, 54, 55]. This is also true for B cells and nonprofessional APCs such as epithelial cells [23, 56]. As discussed above, there is strong evidence to support a role for DCs in mediating antigen presentation during oral tolerance. Nevertheless, the rapidly evolving field of DCs biology has demonstrated that DCs can be divided into several subsets based on their ontogeny and functions. DCs are firstly subclassified into two subsets: classical DCs (that we will refer to as cDCs thereafter) and pDCs (plasmacytoid DCs) [57]. Although pDCs have been reported to be involved in oral tolerance, their role and mechanisms of action remain poorly understood [58]. Classical DCs are further divided into cDC1s and cDC2s. Although cDC1s are generally CD103^+^CD11b^−^XCR1 (X-C motif chemokine receptor 1)^+^, cDC2s are usually classified as CD11b^+^CD103^−^XCR1^−^. In the intestinal system, these cDCs subsets also encompass migratory and resident DCs (both for cDC1s and cDC2s). Although both cDC1s and cDC2s have been linked to oral tolerance induction [44], recent technological advances have shed light on the fact that CD103^+^ cDC1s play a key role in inducing oral tolerance. One of these advances has been the better characterization of DCs subsets and the generation of different strains of mice to target them. Another has been the development of a new technique called LIPSTIC (Labeling Immune Partnerships by SorTagging Intercellular Contacts) [59]. This technique has been developed by the laboratory of Gabriel Victora and has been used to allow the identification of interactions between DCs and CD4 T cells by using the CD40-CD40L interactions (it has later been refined to allow characterization of universal interactions between cell types [60]). The LIPSTIC technique has then been used by the Mucida lab to identify the antigen-presenting cell subsets that interact with T cells during oral tolerance to food. They have notably been able to show that while both cDC1s and cDC2s can interact with T cells, only cDC1s can induce Treg differentiation [61]. They have demonstrated that ablation of MHC-II (Major Histocompatibility Complex class II) expression on cDC1s by using Clec9a (C-type lectin domain family 9 member A)-cre mice resulted in decreased Treg induction [62]. While the deletion of MHC-II on cDC2s using a Zeb2 (Zinc Finger E-Box Binding Homeobox 2) enhancer triple mutant (in which cDC2s development is impaired) did not affect this process [63]. Nevertheless, the effect observed in this study does not seem to completely explain the paradigm that only cDC1s could prime Tregs generation. Indeed, recent multiple independent studies have demonstrated that other types of APCs such as RORγt-APCs also play a role in this induction. We will therefore discuss the functions of RORγt-APCs and how they could regulate oral tolerance induction.

## 3 | RORγt-APCs: Discovery, Diversity and Functions

RORγt-APCs have been described quite recently compared to other types of APCs (for more details see the compelling review by Abramson et al. [19]). The first type of RORγt-APC was described by the Sonnenburg group a decade ago in a seminal paper describing the expression of MHC-II in ILC3s (Type 3 Innate Lymphoid cells) expressing RORγt [64] (Table 1). They went on to discover that these MHC-II ILC3s regulate CD4 T cell responses to enforce tolerance to microbiota and limit the development of intestinal inflammation. This group and others went on to describe in more detail their development and functions in several contexts [65–67] (see below). Ensuing research have demonstrated that other subsets of RORγt-APCs arise as well. It has been reported by several groups that some APCs could express the transcription factor AIRE (Autoimmune Regulator) outside of the thymus, in secondary lymphoid organs [62, 68–71]. Their exact identity and developmental trajectory remain to be completely identified because several groups have reported similarities but also differences among these cells. Nevertheless, these APCs all share antigen presenting capacities and the co-expression of RORγt and AIRE and therefore have been grouped as RORγt-eTACs. Finally, a third type of RORγt-presenting cells has been recently described by the Brown group [72]. These cells, termed Thetis cells, do not express AIRE nor certain markers associated with ILC3s (such as CXCR6 (C-X-C Motif Chemokine Receptor 6) and IL7R (Interleukin 7 Receptor) and resemble more classical DCs.

The function of RORγt MHC-II ILC3s has now been described in more detail (Table 1). The Sonnenberg group has demonstrated that the expression of MHC-II on RORγt-ILC3s restrains the expansion of microbiota-specific T cells via sequestration of pro-survival factors such as IL-2. This resulted in the capacity of these cells to decrease the levels of intestinal inflammation [66]. In a follow-up study, they also showed that RORγt in ILC3s was regulating the expression of the transcription factor Zbtb46 (Zinc Finger And BTB Domain Containing 46) (classically known to be expressed on cDCs) and that Zbtb46 was a major factor to promote the anti-inflammatory phenotype of these cells [73]. Moreover, these ILC3s were also able to control the T follicular helper cells (Tfh), germinal B cells generation and IgA production [67], which promoted the maintenance of homeostasis to commensals. Confirming this regulatory role, MHCII-ILC3s can also limit the mounting of Th2 and Th17 responses in the lungs, in response to allergens and microbes [74]. Overall, these data suggest that RORγt-ILC3s could have a regulatory phenotype in the intestine and the lungs. Nevertheless, this regulatory role might be tissue-specific as well as context-dependent, because it has been reported that RORγt-ILC3s, in inflammatory contexts, could become activated and prime CD4 T cells which enhance the pathogenesis during multiple sclerosis development [75, 76].

RORγt-eTACs have been demonstrated to promote tolerance. The first report of these cells by the Anderson lab (although without identifying RORγt expression) showed that eTACs (Extrathymic Aire-expressing Cells) regulate the expression of several tissue-specific antigens and can mediate deletional tolerance of autoreactive CD4 T cells that escaped negative selection in the thymus [71]. They also showed that MHC-II-eTACs could induce the anergy of these CD4 T cells in the context of type I diabetes [69, 77]. RORγt-eTACs are also critical for the induction of antigen-specific Th17 cells in response to *Candida Albicans* infection in mucosal tissues [70]. However, due to the recent discoveries that these RORγt-eTACs could encompass different cell populations that need to be better defined, their exact role in many different contexts remains poorly understood.

Despite their relatively recent identification, the light has been shed on the field of RORγt-APCs by the simultaneous publication of three independent papers in the same issue of Nature in 2022. These three papers (from the Sonnenberg, Littman and Brown labs) concluded that RORγt-APCs are capable of inducing microbiota-specific peripheral RORγt-Tregs (Tregs expressing RORγt) and establish tolerance to this microbiota [62, 72, 78], despite some difference in phenotype, differentiation and function among the three RORgt-APCs. Although it was originally thought that RORγt-Tregs might be regulated by DCs, which APCs could actually induce them remained largely unknown. These new studies reported that, instead of classical DCs, RORγt-APCs were responsible for the induction of RORγt-Tregs and tolerance to microbiota. They all showed that MHC-II deletion on RORγt-APCs impaired the generation of RORγt-Tregs, whereas MHC-II expression on cDCs was not required. This process requires CCR7 and needs the activation of TGF-β by integrins (especially Integrin α_v_β_8_). However, these studies diverged on the key cell type responsible for the induction of RORγt-Tregs, because they implicated that either MHC-II-ILC3s or RORγt-eTACS (or Janus cells) or Thetis cells (all expressing RORγt) were responsible for this phenomenon. Although it is clear that the RORγt-ILC3s are a specific subset of cells, the scarcity of the different populations of RORγt-eTACs and Thetis cells I-IV at adult age made it difficult to fully appreciate their phenotype. Moreover, it is to be noted that the Brown lab characterized the Thetis cells at an early age (3 weeks of age vs. adult mice) and there is a likely possibility that some of the RORγt-APCs arise early during development (especially if they regulate tolerance) and decline later or differentiate into different subsets. Another important question will be to decipher the developmental trajectory of these different subsets of RORγt-APCs along the time and which progenitors they develop from. For this purpose, it will be necessary to develop tools that can trace and target these cells more specifically. Nevertheless, a subsequent analysis of these three datasets by the Brown lab have attempted to reconcile these findings [79]. By reanalyzing the single cell RNAseq data from these three papers, they confirmed the existence of RORγt-ILC3s, as expected. But the authors also observed that in all databases the different clusters of RORγt-eTACs and Thetis cells described were also present. They notably showed that RORγt-eTACs I, Janus cells I and Thetis cells I mapped together suggesting a unique cell population. They further demonstrated that RORγt-eTACs II, Janus cells II and Thetis cells II-IV share mixed signatures suggesting comparable (between the studies) but mixed cell populations. Interestingly, a recent publication confirmed the presence of these different subsets of RORγt-APCs in several tissues both in human and mouse. They also suggested that these different subsets are functionally versatile and could also have different functions in different contexts [80].

Although these three major subsets of APCs share in common their expression of RORγt and their capacity to present antigens and to some extent their possible roles in inducing tolerance, these RORγt^+^APCs have been reported to differ in several aspects, including their development, phenotype and functions. The development of ILC3s in general is more defined. It appears that conventional ILC3s develop from the common helper innate lymphoid cell precursor (or CHILP) via PLZF (Promyelocytic Leukemia Zinc Finger protein) expression, while LTi-like ILC3s are derived from a unique LTi progenitor [81]. Therefore, both subsets of ILC3s are of lymphoid origin. The origin of RORγt-eTACs is still incompletely understood. Although they share some similarities with classical DCs, they are not marked by a Clec9a-fate mapping reporter demonstrating that they do not develop from the cDC lineage [62]. They have been reported to have a lymphoid morphology and to be traced by an Il7R-fate mapping reporter, suggesting that they might be of lymphoid origin. Moreover, stimulation of ILC3s with RANKL (Receptor Activator of NF-κB Ligand) has the capacity to induce AIRE expression in these cells suggesting (although likely not a major mechanism) that some interconversion could occur between ILC3s and RORγt-eTACs [68]. Nevertheless, a recent report by the Gardner lab has shown that *Il7R* KO or RAG2 (Recombination Activating Gene 2)/IL2RG (IL2 Receptor Gamma) KO bone marrow precursors (both lacking ILCs and lymphoid cells) had a similar capacity than WT mice to generate RORγt-eTACs. They observed that RORγt-eTACs were not labeled with an Ms4a3 lineage tracer suggesting that they are not of monocytic origin, and only minorly by a Clec9a tracer confirming that they are not arising from the DC lineage. However, they reported that most RORγt-eTACs are marked by Cxc3cr1 and Zbtb46 fate mapping reporter mice, suggesting that they are of myeloid origin [82]. Given the reported different sub-populations inside the RORγt-eTACs, it is conceivable that some subsets arise from a lymphoid progenitor (e.g. the ILC3-like cells) and others from a myeloid progenitor (e.g. the Janus and Thetis cells). Finally, it has been shown by the Colonna lab that DC-like Thetis cells arise from lymphoid progenitors but fail to develop from myeloid progenitors emphasizing their lymphoid origin [83]. Nevertheless, the exact mechanisms that lead to the development of these different subsets of RORγt-APCs remain to be fully defined. As for their phenotypic markers, these three subsets of RORγt-APCs can be distinguished by the following markers (in addition to common markers such as these highlighted in Table 1): RORγt-ILC3s express IL7R, CXCR6 but lack AIRE expression; RORγt-eTACs express AIRE but lack CXCR6 and IL7R; Thetis cells lack AIRE, CXCR6 and IL7R but express CD11b, CD11c, ITGB8 and PRDM16 (PR/SET Domain 16) [19, 83, 84]. Finally, although these cells share the capacity to induce tolerance to microbiota and food antigens divergent functions have been reported in some contexts. While TC cells have only been reported to be tolerogenic, RORγt-eTACs have also been shown to promote the generation of *Candida albicans*-specific Th17 cells and their capacity to control infection [70]. Moreover, MHCII-ILC3s have also been reported to prime CD4 T cells and promote neuroinflammation in the context of multiple sclerosis, suggesting a possible dual role depending on their environment [76]. Overall, the current literature underlies some common functions between RORγt-APCs but also some possible divergent roles depend on the context. It is also important to note that due to the recent discoveries of these cells, it is likely that their functions go beyond what is currently described and will be uncovered in the future.

In addition to the diversity of RORγt-APCs, one should also try to understand their roles in the gut in comparison to cDCs, which is currently believed to be the main subset responsible for the induction of oral tolerance. As discussed above, it would seem that DCs and RORγt-APCs have a different ontogeny. Indeed, DCs require a developmental stage through a Clec9a-positive progenitor, which seem to be dispensable for RORγt-APCs development [57, 82]. ILC3s and RORγt-DCs (in contrary to RORγt-eTACs) both come from lymphoid progenitors, and FLT3L (FMS-like tyrosine kinase 3 ligand) seem to be important to induce their differentiation, similarly to cDCs [83, 85, 86]. Nevertheless, it remains to be determined if RORγt-APCs can arise in FLT3L or CD135 (the receptor for FLT3L) deficient mice. It has also been shown that GM-CSF (Granulocyte-macrophage colony-stimulating factor) can promote the development of cDC1s [87] and it would be interesting to understand whether it plays any role in RORγt-APCs development and functions. It would also be interesting to understand whether, similar to cDCs, different waves of RORγt-APCs along development could come from different precursors. In addition, cDCs require CXCR4 to egress the bone-marrow and the action of several chemokines to migrate to different tissues, but whether similar mechanisms occur for RORγt-APCs migration occur (besides a possible role for CCR7) remains unknown [88]. It has been shown that in most mouse tissues, cDCs lifespan is around 10–14 days suggesting a high rate of replacement [89]. Although the turnaround of RORγt-APCs is currently unknown one could speculate that they possess a similar lifespan but this remains to be demonstrated. Moreover, the functions of cDCs are largely well described in several contexts (both in and outside the gut), while the determination of the roles of RORγt-APCs is still in its infancy. It is quite intriguing that, in addition to a well described role in inducing oral tolerance, cDCs also play important roles in regulating anti-pathogens responses. They can sense bacteria in the lumen of the intestine [90], can respond to DAMPs (Damage-Associated Molecular Patterns) and PAMPs (Pathogen-Associated Molecular Patterns) (notably via PRR (Pattern recognition receptors) sensing) and initiate several types of anti-viral and anti-bacterial responses [91–93]. Moreover, they also have a high capacity to migrate into LNs and process antigens. cDCs also are implicated in several diseases such as cancer [94] (with cDC1s having a more protective role than cDC2s) or autoimmune diseases (such as IBD (Inflammatory Bowel Disease) [95]). It is indicated that cDC1s have a high capacity to promote cross-presentation of antigens to CD8 T cells while cDC2s activate preferentially CD4 T cells [57]. Whether RORγt-APCs perform most of these aforementioned functions (besides induction of tolerance) remain to be addressed. Nevertheless, and as described above, RORγt-ILC3s can modulate CD4 T cells (either positively or negatively depending on the context) suggesting a role beyond the induction of tolerance [76]. Moreover, RORγt-eTACs have the capacity to promote the generation of *Candida Albicans*-specific Th17 and to promote protection against infection, a role that has also been ascribed to cDCs before [70]. In addition, RORγt-eTACs share the capacity with cDCs to migrate in the mLNs in response to the TLR7/8 (Toll like receptor 7/8) ligand R848 [82]. These data suggest that RORγt-APCs might have similar roles than cDCs in some contexts, nevertheless, whether RORγt-APCs and cDCs share a division of labor, cooperate together or might have redundant functions remains to be discovered. An important factor to take into account regarding their possible roles is the timing of their appearance during development. For example, RORγt-DCs seem to arise early after weaning, while cDCs seem to develop during embryogenesis [72]. It would also appear that the timing of intervention of these cells could be complementary to ensure that a process is maintained over time [61]. Finally, the question of the localization of these different cell subsets is likely to explain some of the functions of these cells. For example, cDC1s and TCs seem to have a paracortical location in the LNs, while cDC2s and ILC3s are localized in the interfollicular regions [62, 72, 96]. This localization is likely to be important for their function because it reflects the positioning of CD4 and CD8 T cells in the LNs and because it has been reported that, due to their localization, cDC2s are faster at acquiring antigens than cDC1s [97]. Therefore, we speculate that the following years will be the subject of an intense investigation in determining how cDCs and the different subsets of RORγt-APCs regulate immune responses and homeostasis in different contexts, especially in mucosal tolerance. We will now describe in more extent the roles of these cells in regulating oral tolerance.

## 4 | RORγt-APCs, Key Players in Oral Tolerance?

**FIGURE 2 |:**
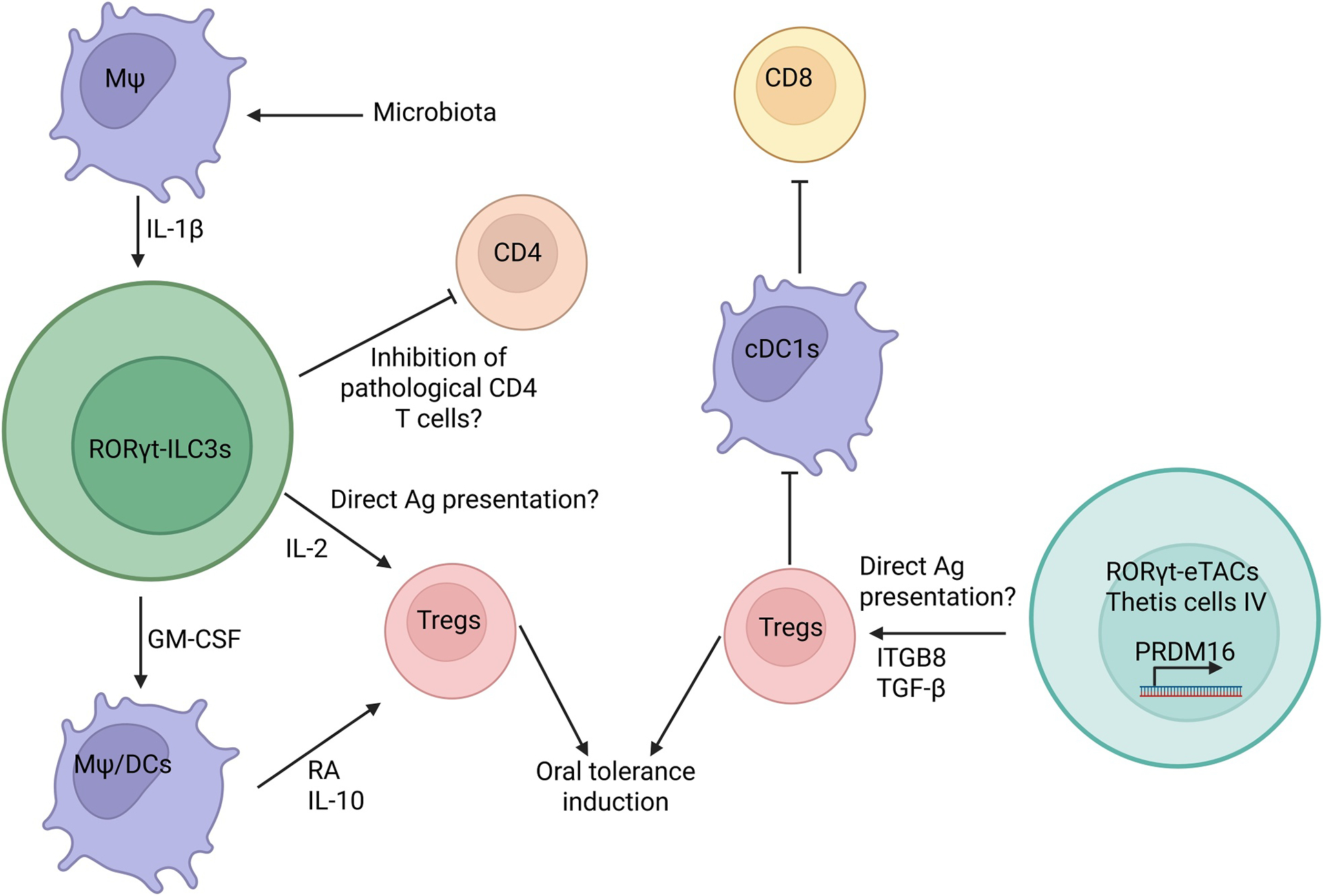
Regulation of oral tolerance by RORγt-APCs. RORγt-ILC3s are capable of sensing IL-1β produced by macrophages in response to microbiota. This has two main effects on their capacity to induce Tregs. The first is through a direct effect via production of IL-2. The second one is indirect, via production of GM-CSF which educates macrophages and DCs. They will therefore produce, in turn, RA and IL-10 to promote the induction of Tregs, which leads to the induction of oral tolerance. It is also possible (although not directly demonstrated) that RORγt-ILC3s could present antigens directly to T cells. This could promote the generation of Tregs and restrains the generation of pathogenic CD4 T cells. In the other hand, RORγt-eTACs and Thetis cells IV seem to be able to directly present antigens to Tregs to induce their generation. This process involves TGF-β and its activator ITGB8, and is dependent on the transcription factor PRDM16. These induced Tregs could keep in check cDC1s, which later on will result in the modulation of CD8 T cells, another way to modulate oral tolerance cellular circuits. DCs, Dendritic Cells; ITGB8, Integrin beta-8; Mψ, Macrophages; PRDM16, PR domain-containing 16; RA, Retinoic Acid; TGF-β, Transforming Growth Factor Beta; Tregs, Regulatory T cells.

A certain number of evidence in previously published papers showed that RORγt-APCs seem to have a regulatory role in different contexts, and especially in their ability to induce RORγt-Tregs and promote tolerance to microbiota in the gut. It has also been noted that the mechanisms by which oral tolerance to food antigens is mediated are rather unclear, especially regarding the subsets of APCs that mediate this effect. It was therefore tempting to hypothesize that RORγt-APCs could play a role in the establishment of oral tolerance. The first reports of a possible role of RORγt-APCs in oral tolerance came some years ago from the Merad and Sonnenberg groups, in which they studied the role of RORγt-ILC3s (Figure 2). The Merad group first showed that, RORγt-ILC3s in the gut were able to produce GM-CSF, a factor necessary for the homeostatic function of macrophages [48]. Therefore, the disturbance of GM-CSF production by ILC3s led to a decreased generation of Tregs in the colon and small intestine, as well as a decrease in oral tolerance notably marked by increased development of delayed-type I hypersensitivity. Interestingly, a feedback loop is responsible for the production of GM-CSF by ILC3s, in which macrophages, in response to microbiota, produce IL-1β which is responsible for the production of GM-CSF. A few years later, the Sonnenberg lab reported a different mechanism by which RORγt-ILC3s promote the induction of Tregs and oral tolerance [49]. They demonstrated that macrophages production of IL-1β in response to microbiota could drive the production of IL-2 by ILC3s. Interestingly, the production of IL-2 by these cells (but not by CD4 T cells) was crucial for Tregs maintenance in the intestine and the establishment of oral tolerance.

More recently, and as discussed briefly above, the use of the LIPSTIC method allowed the determination of the cell types involved in the interaction with T cells in the context of oral tolerance establishment [61]. Although the authors reported that cDC1s are important, the deletion of MHC-II on cDC1s only accounted for a part of Tregs induction, suggesting that other cells could play a role. They notably reported that, early after OVA administration, a subset of Thetis cells and RORγt-ILC3s could interact with CD4 T cells. Although overall cDCs seemed to be the major cell type to interact with T cells, RORγt-APCS composed the majority of these cells at 0–2h, and remained substantial until 6h. In this paper, the authors, however, did not address the functional role of RORγt-APCs in oral tolerance establishment.

Nevertheless, several articles from the Brown, Colonna, Gardner, Littman and Kedmi labs tried to functionally address the possible role of RORγt-APCS in the establishment of oral tolerance [82–84, 98, 99]. The Brown lab showed that RORγt-Thetis cells were able to take up antigens in the mLN, induce regulatory T cells (in a ITGB8-dependent manner) and promote oral tolerance [98]. Surprisingly, and contrary to Canesso et al., they reported that cDC1s were promoting the induction of Th1 and memory T cells. Interestingly, the Kedmi lab reported similar findings [99]. They determined that RORγt-APCs were able to induce Tregs in response to dietary antigens while cDC1s were dispensable. However, they suggest that once Tregs are generated, these Tregs are able to educate the cDC1s to limit the expansion of CD8αβ T cells. These CD8 T cells, although tolerogenic to food antigens, were protective against a pathogen in the context of *Listeria monocytogenes* infection. Therefore, this article defines a complex cellular circuit between RORγt-APCS, Tregs and CD4 T cells, cDC1s and CD8 T cells that offers protection during infections, while maintaining tolerance toward oral food antigens. As the authors point out, the mechanisms involved in this circuit will need to be further elucidated. The Littman group independently corroborated these findings and also showed that RORγt-APCS are crucial for oral tolerance establishment [84]. Interestingly, they also went on to better characterize these cells at the molecular level. They showed that RORγt-APCs rely on a unique *cis*-regulatory element in the Rorc gene locus (Rorc(t) + 7kb) which is dispensable for T cell differentiation. The deletion of this regulatory element impaired oral tolerance (and microbiota-induced Tregs) and led to the induction of Th2 cells instead of Tregs during asthma development. They also identified the transcription factor PRDM16 as a unique transcription factor expressed by tolerogenic RORγt-APCs in both mouse and human, defining a potential target to specifically modulate RORγt-APCS. Indeed, deletion of *Prdm16* in these cells abrogated the induction of oral tolerance. Quite importantly, a similar paper was just published by the Colonna lab that confirmed most of these findings [83]. They observed that both RORγt-ILC3s and RORγt-DCs (which includes Thetis and RORγt-eTACs) depend on the Rorc(t) + 7kb locus for their development. Therefore, Rorc(t) + 7kb mutated mice have a deficit in mounting oral tolerance. They also confirmed that PRDM16 is a specific marker for RORγt-APCs, but also identified Gms38411 (a long noncoding antisense RNA) as a specific marker of RORγt-APCs, which expression follows RORγt expression pattern. They therefore used a new reporter mouse line (Gms38411^iCre-hCD2^R26^tdTomato^) that allowed further identification of RORγt-APCs. Their data notably suggest that RORγt-DCs are a subset of migratory cDC2s that originate from a lymphoid progenitor (which could fit a previous report of cDC2s expressing RORγt [100]). They went a step further by identifying a specific marker of ILC3s, Serinc2 (Serine Incorporator 2, a transmembrane protein involved in lipid metabolism), and by generating Serinc2 ^iCre^-Rorc^fl/fl^ mice. They showed that these mice do not have any defects in generating Tregs during homeostasis, suggesting that ILC3s could be dispensable to generate Tregs, which is opposite to previous reports as we discussed earlier. Nevertheless, it has to be noted that here, the authors depleted RORγt in ILC3s but it is still unknown whether RORγt is actually important for their function or only a marker to identify them. Moreover, they did not test whether Serinc2^iCre^-Rorc^fl/fl^ mice are deficient in mounting oral tolerance or if the effect observed is only at steady-state. Finally, and quite interestingly, Rorc(t) + 7kb mutant mice already have a defect in inducing Tregs (and increased Th2) at steady-state, in an environment where peripheral Tregs (especially in specific-pathogen mice) should not encounter many antigens presented by APCs. This could suggest that RORγt-APCS could be involved in other processes than directly presenting antigens to T cells during oral tolerance.

Although many advances have been made over the recent years, it is still rather unclear what are the cellular and molecular circuits involved in the mechanisms of oral tolerance [18]. The recent reports about RORγt-APCs role in oral tolerance development suggest that these cells are playing a key role in this process, but at which step and through which mechanisms is still unclear. Moreover, some of these reports diverge on whether cDC1s are the main APCs to present antigens to Tregs. It has to be noted that several factors could explain this apparent discrepancy. First, the mouse age seems to be a critical factor, especially since Thetis cells seem to be predominant at early age but could be less important during later development. It is therefore possible to think that some cell types play a critical role at some developmental stage and that other cells take the reins later on. More specifically, while most articles used adult mice, the studies performed by the Brown lab used mice at postnatal age [72]. It is interesting to note that while Canesso et al., observed an effect of MHC-II deficiency in cDC1s, this effect was not observed by Cabric et al., [61, 101]. This could be explained by the age of the mice used in their respective studies. Notably, when they looked at early age, Canesso et al., found that RORγt-APCs interacted with OT-II T cells at a much higher level than later on, after which cDCs seemed to take the rein in interacting with T cells. An important question that remained unanswered is whether aged mice have a deficit in oral tolerance induction and whether any of the RORγt-APCs subsets are affected during aging. Another important factor to consider is microbiota. It has been demonstrated that RORγt-APCs and cDCs are all modulated by the microbiota and it is therefore likely that different conditions could affect the outcome of these studies. It is also possible that the context in which oral tolerance is induced matters. For example, if the mice are subjected to infections or inflammation/autoimmunity, we could imagine a division of labor between different APCs, as well as a shift in their capacity to induce oral tolerance from one to another. It is also important to note that RORγt-APCs could impact different steps of oral tolerance induction. It is also possible to envision that some APCs could regulate the antigen uptake, some specify its presentation to CD4 or CD8 T cells (or their anergy, differentiation to different T helper subsets (such as Th1, Th2 or Th17)), some maintain the survival or migration of Tregs or some perpetuate the consequence of Tregs activation. For example, it would appear that the RORγt-ILC3s subset regulates oral tolerance by regulating IL-2 production and macrophage functions, rather than by directly interacting with T cells. It is also important to note here that the role of RORγt-ILC3s in directly presenting the antigens to T cells (notably via specific deletion of MHC-II in RORγt-ILC3s) has not been addressed yet. Another question to be answered is whether some APCs are able to regulate oral tolerance that affects a particular tissue (e.g., skin vs. lungs) as well as to a particular type of antigen (such as a protein vs. a peptide vs. a hapten, or proteins of different structures e.g.). While some studies have been using a model of type I delayed hypersensitivity, some others have been using a model of allergic airway (and one model of food allergy). Although it did not seem to impact the outcome of the studies, it would be interesting to understand if different RORγt-APCs contribute to a different degree in the observed effects. Here, it is important to note that most studies have been performed with OT-II transgenic T cells and in response to ovalbumin, which (although a useful tool) is likely to be largely unrepresentative of physiological oral tolerance induction [102]. Although all the studies cited above have used ovalbumin as an antigen, it is conceivable that different subsets of RORγt-APCs could regulate oral tolerance to different antigens, or even to different concentrations of antigens. Another future challenge will be to fully characterize the different subsets of RORγt-APCs and decipher their development/differentiation, and to develop tools to specifically modulate each cell subtypes. For example, earlier studies demonstrating a role of RORγt-ILC3s in oral tolerance development have used several different models including global KO mice (Csf2 KO or Rag2/IL2RG KO, coupled or not with ILC3s transfer) or Ncr1-cre mice (which can target NK cells and most ILCs), but did not specifically targeted ILC3s nor deleted MHC-II in these cells, suggesting a possible contamination of these cells as well as the possibility that they are not directly involved in antigen presentation but in other steps of oral tolerance [48, 49]. Interestingly, the Colonna group generated a new mouse model to target ILC3s, namely Serinc2^icre^ mice, which they used to demonstrate that the phenotype observed by them and Fu et al., by generating Rorc(t) + 7kb mutant mice is likely not due to ILC3s [83]. Nevertheless, they analyzed the mice at steady-state and in a *Clostridium difficile* model and did not study these mice in an oral tolerance model. Another interesting report from the Gardner lab, also confirmed the importance of RORγt-APCs in oral tolerance [82]. They pinpointed a role for RORγt-eTACs by using a combination of mixed bone-marrow chimeras experiments and the use of AIRE-DTR mice to demonstrate the importance of RORγt-eTACs in inducing RORγt-Tregs but showed that they are likely not important to induce RORγt-negative Tregs. Although it clearly appears that RORγt-APCs play a critical role in inducing oral tolerance, it is likely that the different mouse models used in these studies could at least partly explain the apparent discrepancies. Therefore, it will be important in the future to develop mouse models to specifically delete (and target) the different subsets of RORγt-APCs and cDCs in order to decipher which exact population(s) (and how) mediate the observed effects.

## 5 | Translational Relevance of RORγt-APCs

Finally, what is the exact function of these different APCs, how they are regulated and how they could be harnessed to promote oral tolerance in humans remains to be fully determined. It is of particular importance since feeding oral antigens could be a way to develop new prevention and therapeutics in the context of autoimmune diseases in clinical settings. Importantly, the existence of RORγt-APCs has also been confirmed in humans. The group of Dr. Sonnenberg identified a decrease in frequency of both ILC3s and Tregs in the human intestine of patients with Crohn's disease [49]. This was notably associated with a decreased production of IL-2 by these ILC3s. They also later on confirmed these findings by performing scRNAseq on IBD patients, where they could also observed a decrease in RORγt-Tregs as well [62]. More recently, several groups have identified the presence of RORγt-DCs/TCs in several human organs. Analysis of several databases and newly generated data by the Colonna, Littman and Schraml labs have shown that RORγt-DCs can be identified in the mLN, lamina propria, spleen and human tonsils [80, 83, 84]. They identified this population of cells as co-expressing RORγt and PRDM16. Nevertheless, they could not identify it in the bone marrow suggesting that, in human too, these cells might develop at early age. They also observed by analyzing scATACseq data that the RORγt 7kb locus was open in these cells, suggesting that they also depend, as in mice, on this locus to develop. Significantly, the Colonna lab was also able to generate RORγt-APCs (and notably DCs) from human tonsils and showed that they are capable of stimulating T cells. The existence of these RORγt-APCs in humans is of great importance because they could be targeted to develop new therapeutics in the context of allergy, asthma and autoimmune diseases. It is important to note that PRDM16 polymorphisms are associated with the development of asthma, allergy, arthritis or IBD [103–106]. In addition, it is still unknown whether RORγt-DCs/TCs/eTACs are also dysregulated during human diseases, but the fact that RORγt-ILC3s are, suggests that they might be dysfunctional as well. Therefore, it will be of the utmost importance to fully characterize the development, phenotype, function and dysregulation of RORγt-APCs during human health and disease. This could help better understand how immune-mediated diseases such as food allergy, asthma, or autoimmune diseases (such as IBD) develop. For example, it can be hypothesized that peanut allergy could be better counteracted by promoting feeding in infants due to the presence of RORγt-DCs in early developmental stages [107]. It could also help target these cells to modulate these diseases. The possibility to generate RORγt-APCs from human cell culture notably holds some promises to use them in the future as a cellular therapy. While modulating oral tolerance had held great promises in pre-clinical models, this had always been difficult to translate to humans [108–111]. The discovery of RORγt-APCs could therefore be the missing link to help us target oral tolerance in human diseases.

## 6 | Concluding Remarks

Oral tolerance is a critical mechanism of survival in humans and mammals to prevent the development of autoimmune pathologies. Despite recent advances, the molecular and cellular mechanisms by which oral tolerance is established still remain largely unclear. The recent discovery and characterization of several subsets of APCs, especially RORγt-APCs, shed a new and exciting light on this process and at the same time warrant further investigation. The better understanding and new advances in this field will not only help our understanding of the development of diseases such as allergies, asthma and chronic inflammation and autoimmunity, but also facilitate promoting the development of new therapeutics for the treatment of related human diseases.

## Figures and Tables

**TABLE 1 |:** Key markers and functions of RORγt-APCs.

Cell subset	Markers	Function
RORγt-ILC3s	CCR6^+^IL7R^+^CXCR6^+^CCR7^+^MHC-II^+^CD80/86^+/−^RORγt^+^ZBTB46^+^	Inflammatory ILC3s (expressing CD80/86) induce Th17 cell expansion, T cell reactivation in the CNS and promote inflammation [75, 76]. LTi-like ILC3s inhibit T cells activation and promote the induction of microbiota-sepcific RORγt-Tregs [62, 64, 66, 67, 73, 74, 78].
RORγt-eTACs	CCR6^+^IL7R^−/low^CXCR6^−^CCR7^+/^low MHC-II^+^CD80/86^+/−^RORγt^+^ZBTB46^+^Aire^+/low^PRDM16^+/?^	Promote Th17 expansion (Aire + ILC3s like) [70]. Restrain T cell activation [71, 77]. Promote the generation of RORγt-Tregs in response to microbiota [78].
Thetis cells IV	CCR6^+^IL7R^−/low^CXCR6^−^CCR7^+/low^CD11b^+^CD11c^+^MHC-II^+^CD80/86^+/−^RORγt^+^ZBTB46^+/low^PRDM16^+^	Promote the induction of microbiota-sepcific RORγt-Tregs [72].

## Data Availability

Data sharing is not applicable to this article as no new data were created or analyzed in this study.
